# A Novel Role for DOC2B in Ameliorating Palmitate-Induced Glucose Uptake Dysfunction in Skeletal Muscle Cells via a Mechanism Involving β-AR Agonism and Cofilin

**DOI:** 10.3390/ijms25010137

**Published:** 2023-12-21

**Authors:** Jinhee Hwang, Rekha Balakrishnan, Eunjin Oh, Rajakrishnan Veluthakal, Debbie C. Thurmond

**Affiliations:** 1 Department of Molecular and Cellular Endocrinology, Arthur Riggs Diabetes and Metabolism Research Institute, Beckman Research Institute at City of Hope, Duarte, CA 91010, USA; jihwnag@korea.ac.kr (J.H.); rbalakrishnan@coh.org (R.B.); euoh@coh.org (E.O.); rveluthakal@coh.org (R.V.); 2 Department of Food and Biotechnology, College of Science and Technology, Korea University, Sejong 30019, Republic of Korea

**Keywords:** DOC2B, β-adrenergic receptor, insulin resistance, skeletal muscle, GLUT4, glucose uptake, type 2 diabetes, palmitate, lipotoxic stress, cofilin

## Abstract

Diet-related lipotoxic stress is a significant driver of skeletal muscle insulin resistance (IR) and type 2 diabetes (T2D) onset. β_2_-adrenergic receptor (β-AR) agonism promotes insulin sensitivity in vivo under lipotoxic stress conditions. Here, we established an in vitro paradigm of lipotoxic stress using palmitate (Palm) in rat skeletal muscle cells to determine if β-AR agonism could cooperate with double C-2-like domain beta (DOC2B) enrichment to promote skeletal muscle insulin sensitivity under Palm-stress conditions. Previously, human T2D skeletal muscles were shown to be deficient for DOC2B, and DOC2B enrichment resisted IR in vivo. Our Palm-stress paradigm induced IR and β-AR resistance, reduced DOC2B protein levels, triggered cytoskeletal cofilin phosphorylation, and reduced GLUT4 translocation to the plasma membrane (PM). By enhancing DOC2B levels in rat skeletal muscle, we showed that the deleterious effects of palmitate exposure upon cofilin, insulin, and β-AR-stimulated GLUT4 trafficking to the PM and glucose uptake were preventable. In conclusion, we revealed a useful in vitro paradigm of Palm-induced stress to test for factors that can prevent/reverse skeletal muscle dysfunctions related to obesity/pre-T2D. Discerning strategies to enrich DOC2B and promote β-AR agonism can resist skeletal muscle IR and halt progression to T2D.

## 1. Introduction

Glucose uptake by skeletal muscle plays a crucial role in preventing postprandial hyperglycemia. Nonetheless, the onset of insulin resistance induced by a Western-style diet is accompanied by elevated levels of lipids, particularly palmitic acid (hexadecanoic acid, C16:0), which hinders insulin-stimulated glucose uptake and impairs skeletal muscle function, ultimately contributing to the pathogenesis of Type 2 diabetes (T2D) [1,2,3]. Therefore, strategies that can increase glucose uptake in skeletal muscle under insulin-resistant conditions are warranted as therapeutics for mitigating the rise in prediabetes and T2D.

The uptake of glucose from the circulation into skeletal muscle requires both insulin signaling and glucose transporter-4 (GLUT4) vesicle trafficking. The binding of insulin to the insulin receptors induces autophosphorylation of the insulin receptor and subsequent phosphorylation of the insulin receptor substrate-1 (IRS-1), which in turn activates phosphatidylinositol-3-kinase (PI3K). The downstream signaling pathway of PI3K bifurcates into two parallel insulin signaling arms, each playing a crucial role in promoting GLUT4 vesicle translocation. One arm involves PI3K-Akt-AS160-Rab GTPase leading to GLUT4 vesicle translocation, while the other arm is composed of PI3K-Rac1 and actin remodeling to promote GLUT4 vesicle mobilization in skeletal muscle [4].

In addition to the well-established insulin signaling pathway that drives glucose uptake in skeletal muscle via GLUT4 vesicle translocation, the insulin-independent signaling pathway involves the β-adrenergic receptor (β-AR), a member of the G-protein-coupled receptors (GPCR) family. β-AR agonists, including isoproterenol, clenbuterol, and BRL-37344, increase glucose uptake in vivo and rat L6 skeletal muscle cells [5,6,7]. Additionally, prolonged treatment with either low or high doses of clenbuterol has been observed to improve glucose and insulin tolerance in rodent models for insulin resistance and T2D [7,8,9,10,11]. Emerging evidence indicates that β-AR signaling enhances glucose uptake in skeletal muscle by triggering insulin-independent GLUT4 vesicle mobilization [5,8]. While the molecular mechanisms underlying the initiation of GLUT4 vesicle mobilization to the plasma membrane (PM) are well-detailed in response to insulin [12,13], less is known about the response to β-AR signaling and the potential for synergy with insulin-dependent signaling. Further, a detailed understanding of the molecular mechanistic dysfunctions caused by exposure to the specific toxic fatty acids affiliated with obesity, pre-T2D, and T2D is needed to pursue strategies that can prevent or reverse skeletal muscle insulin resistance. Double C-2-like domain beta (DOC2B) is essential for GLUT4 vesicle exocytosis in muscle cells [14,15,16]. DOC2B-deficient mice are glucose intolerant due to impaired peripheral insulin sensitivity [17,18]. Indeed, T2D individuals show a deficiency in DOC2B levels in the skeletal muscle [14]. What remains to be known is how DOC2B interacts with β-AR signaling during insulin-stimulated GLUT4 translocation to the PM within the context of lipotoxic stress.

DOC2B is a broadly expressed protein comprising an *N*-terminal Munc13-interacting domain and two tandem C2 domains (C2A and C2B) at the C-terminus [19,20,21]. DOC2B also interacts with components of the cytoskeleton, in a role related to the vesicle mobilization [22,23]. For example, in islet β-cells, DOC2B associates with ERM proteins (ezrin, radixin, and moesin) that interact with the cytoskeleton to trigger insulin granule mobilization [24]. In L6-GLUT4myc skeletal muscle cells, DOC2B interacts with the microtubule protein kinesin light chain 1 (KLC1) to facilitate the GLUT4 vesicle translocation [14]. In addition, skeletal muscle GLUT4 vesicle mobilization requires the Arp2/3 complex and cofilin cycling [25,26,27,28]. While cofilin plays an important role in cytoskeletal remodeling in skeletal muscle cells, connectedness to DOC2B in any cell type has yet to be reported.

In the present study, we established an in vitro paradigm of palmitate (Palm)-induced stress in skeletal muscle cells; palmitate is recognized as a strongly deleterious fatty acid for skeletal muscle function [3,29,30]. Using this Palm-stress paradigm, we interrogated β-AR agonism of insulin-stimulated GLUT4 trafficking and how it relates to DOC2B and cofilin-regulated cytoskeletal remodeling. The present study provides valuable information for strategies that enhance DOC2B levels in skeletal muscle cells to negate the deleterious effects of palmitate exposure upon cofilin and β-AR agonism during insulin-stimulated GLUT4 trafficking to the PM and glucose uptake.

## 2. Results

### 2.1. β2-Adrenergic Receptor (β-AR) Signaling Synergizes with Insulin to Enhance GLUT4 Translocation

**Figure 1:**
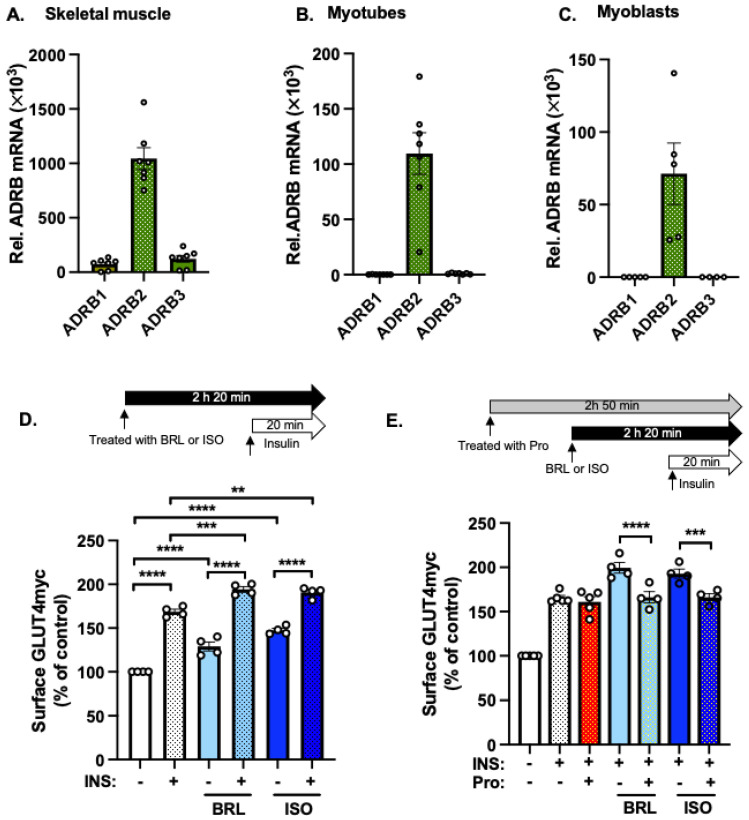
β-AR agonism enhances insulin-stimulated GLUT4 translocation to the PM. (**A**) Analysis of ADRB mRNA family member expression (genes encoding the β-AR family members) relative to Hypoxanthine Phosphoribosyltransferase (HPRT) housekeeping gene in male C57BL/6J mouse quadricep skeletal muscle (*n* = 7 mice). (**B**) ADRB mRNA expression (relative to HPRT housekeeping gene) in L6-GLUT4myc myotubes (*n* = 7 independent passages). (**C**) ADRB mRNA expression (relative to HPRT housekeeping gene) in L6-GLUT4myc myoblasts (*n* = 5 independent passages). (**D**,**E**) The effect of β-AR agonists (BRL or ISO) on PM GLUT4myc accumulation ± insulin (INS) in L6-GLUT4myc myoblasts (*n* = 4 independent passages/condition). In (**E**), cells were pretreated with β-blocker (Pro) prior to β-AR agonist application. The immunofluorescence intensity of PM GLUT4myc was normalized to the fluorescence intensity of the nuclear DNA stain SYTO 60. (**A**–**E**) Data are expressed as mean ± standard error of the mean (SEM). ** *p* < 0.01, *** *p* < 0.001, **** *p* < 0.0001. Green indicates mRNA expression; red indicates the β-AR blocker propranolol treatment; light blue indicates the β-AR agonist BRL treatment; dark blue indicates the β-AR agonist ISO treatment.

Given that glucose uptake in skeletal muscle is stimulated by β-AR, we questioned whether β-AR signaling synergizes with insulin to enhance GLUT4 vesicle translocation. To embark on this, we first examined which β-AR subtypes were predominately expressed in mouse skeletal muscle tissue and rat L6-GLUT4myc cells. The expression of the β_2_-AR mRNA was an order of magnitude higher than that of either β_1_-AR or β_3_-AR mRNA (Figure 1A). In L6-GLUT4myc myotubes (Figure 1B) and L6-GLUT4myc myoblasts (Figure 1C), β_2_-AR mRNA showed high expression, as in primary skeletal muscle, with very low levels of β_1_-AR or β_3_-AR mRNA, supporting the choice of the L6-GLUT4myc cell line as a model for subsequent skeletal muscle β-AR molecular mechanism studies.

To investigate the effect of the simultaneous activation of β_2_-AR and insulin signaling on GLUT4 vesicle translocation, we quantified the GLUT4myc protein accumulation at the PM in non-permeabilized L6-GLUT4myc myoblasts, capitalizing on the exofacial presentation of the myc tag upon GLUT4 vesicle fusion, as previously described [2]. L6-GLUT4myc myoblasts stimulated with insulin (100 nM) alone elicit a ~160% increase in exofacially-exposed GLUT4myc (Figure 1D), as detected using anti-myc staining of unpermeabilized cells, compared with levels in unstimulated cells. By contrast, pre-incubation for 2 h with a β_2_-AR agonist, BRL-37344 (BRL, 10 μM), or with a pan-β-AR agonist (isoproterenol, ISO, 1 μM), followed by 20 min insulin stimulation, further enhanced GLUT4myc accumulation at the PM (Figure 1D), compared with that of insulin or agonists alone. Agonists alone without insulin stimulation also showed a significant increase in GLUT4myc accumulation (Figure 1D). To validate the specificity of the additive effect of the agonists, cells were pretreated with the β-AR blocker propranolol (Pro, 10 μM) for 30 min before the addition of BRL or ISO, and then stimulated with insulin for 20 min. Propanol pre-treated cells exhibited no enhancement with BRL or ISO (Figure 1E). These results suggest that β-AR agonism enhances insulin-stimulated GLUT4 accumulation at the PM.

### 2.2. Palmitate Impairs the β-AR Agonism of Insulin-Stimulated GLUT4 Accumulation at the PM

**Figure 2:**
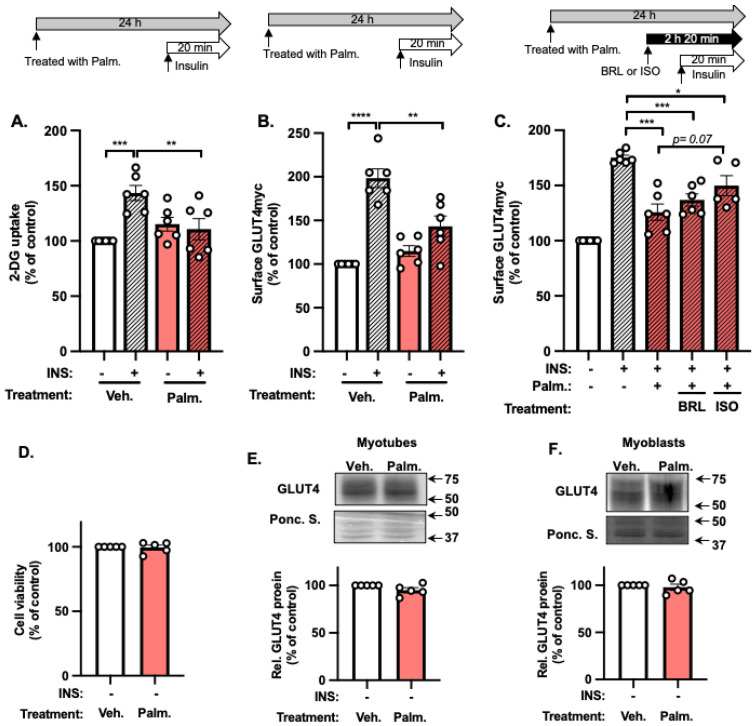
Lipotoxic stress impairs insulin-stimulated- and β-AR agonism-induced-GLUT4 translocation to the PM. (**A**–**C**) Analysis of 2-DG uptake (**A**) and PM GLUT4 levels (**B**) in L6-GLUT4myc myotubes treated with Palm or vehicle (Veh) ± insulin (INS) stimulation. *n* = 6 independent experiments. (**C**) Myoblasts subjected to Palm stress were also incubated with β-AR agonists (BRL or ISO) for 2 h prior to insulin stimulation. *n* = 5–6 independent experiments. The immunofluorescence intensity of PM GLUT4myc was normalized to the fluorescence intensity of the nuclear DNA stain SYTO 60. (**D**) Assessment of cell viability in L6-GLUT4myc myoblasts grown under stated conditions. *n* = 5 independent experiments. (**E**,**F**) Quantification of GLUT4 protein abundance in L6-GLUT4myc myotubes (**E**) and L6-GLUT4myc myoblasts (**F**) grown under stated conditions. *n* = 5 independent experiments. Representative immunoblots are shown. Ponceau S (Ponc. S) staining of the full-length PVDF membrane was used as a loading control. (**A**–**F**) Data are expressed as mean ± SEM. * *p* < 0.05, ** *p* < 0.01, *** *p* < 0.001, **** *p* < 0.0001.

A significant driver of prediabetes and T2D is lipotoxic stress on skeletal muscle cells, which induces insulin resistance. Since β-AR carries the potential to stimulate GLUT4myc accumulation at the PM, it might be able to protect against lipotoxicity-induced insulin resistance. To test this, we first established the paradigm of lipotoxicity, wherein palmitate (Palm)-pre-treatment of myotubes for 24 h decreased insulin-stimulated glucose uptake, as measured by 2-deoxy[1,2-^3^H] glucose (2-DG) uptake (Figure 2A), and impaired insulin-stimulated GLUT4myc accumulation at the PM (Figure 2B). Using this paradigm, we tested whether 2 h treatment with BRL or ISO prior to insulin stimulation (20 min) would reduce the Palm-induced stress. The Palm-induced stress substantially reduced the β-AR enhancement of GLUT4myc accumulation, with no statistically significant improvement afforded by BRL or ISO (Figure 2C). This lack of β-AR amelioration of insulin resistance was not the result of Palm-stress-induced decreases in cell viability (Figure 2D) or total cellular GLUT4 protein abundance (Figure 2E,F). These results pointed to Palm-stress-induced defects in downstream GLUT4 vesicle trafficking.

### 2.3. Palmitate Impairs the Abundance of DOC2B Protein, a Distal Trafficking Protein Required for GLUT4 Translocation

**Figure 3:**
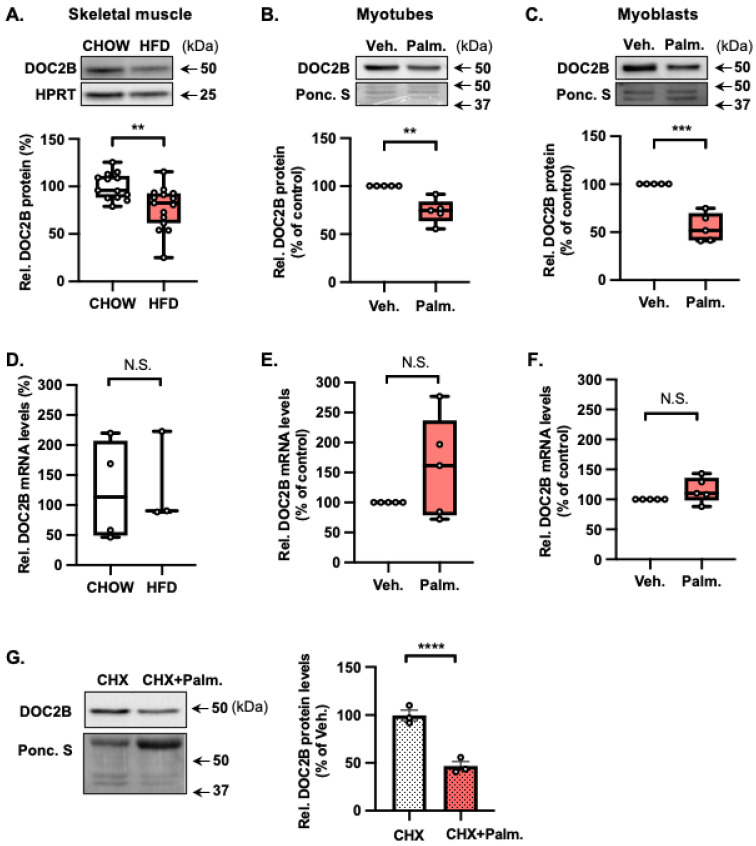
Palm stress impairs DOC2B expression. (**A**) Immunoblot analysis and quantification of DOC2B protein abundance in male C57BL/6J mouse skeletal muscle. Mice were fed an HFD (*n* = 15) or chow diet (*n* = 13). The protein level was normalized to HPRT. (**B**,**C**) Immunoblot analysis and quantification of DOC2B protein abundance in L6-GLUT4myc myotubes (**B**) and myoblasts (**C**) under stated conditions (*n* = 5 independent experiments). Representative immunoblots are shown. Ponceau S (Ponc. S) staining of the full-length PVDF membrane was used as loading control. (**D**) Analysis of *DOC2B* mRNA expression relative to HPRT housekeeping gene in male C57BL/6J mouse skeletal muscle. As in (**A**), mice were fed an HFD (*n* = 3) or chow diet (*n* = 4). (**E**,**F**) Analysis of *DOC2B* mRNA expression relative to *HPRT* housekeeping gene in L6-GLUT4myc myotubes (**E**) and L6-GLUT4myc myoblasts (**F**) under stated conditions (*n* = 5 independent experiments). (**G**) Immunoblot analysis and quantification of DOC2B protein abundance in L6-GLUT4myc myoblasts under stated conditions (*n* = 3 independent experiments). A representative immunoblot is shown. Ponceau S staining of the full-length PVDF membrane was used as a loading control. (**A**–**G**) Data are expressed as mean ± SEM. N.S.; not significant, ** *p* < 0.01, *** *p* < 0.001, **** *p* < 0.0001.

We sought to investigate the mechanism(s) by which lipotoxic stress impairs both the insulin and β-AR signaling-stimulated GLUT4 accumulation at the PM. T2D skeletal muscle is deficient in DOC2B [14], a protein shown to be essential for GLUT4 vesicle exocytosis in muscle cells [14,15,16]. Given this, we questioned whether Palm-based lipotoxicity impacted skeletal muscle DOC2B protein levels. Indeed, a significant reduction was observed in skeletal muscle DOC2B protein abundance in male C57BL/6J mice fed a Palm-based high-fat diet (HFD; 45% calories from fat) for 13 weeks compared to chow-fed mice (Figure 3A). This loss of DOC2B protein was recapitulated in L6-GLUT4myc myotubes (Figure 3B) and myoblasts (Figure 3C) versus vehicle control following 24 h exposure to 200 µM Palm. However, Palm stress did not impact DOC2B mRNA levels in skeletal muscle (Figure 3D), L6 myotubes (Figure 3E), or L6 myoblasts (Figure 3F). To investigate the mechanism(s) underlying how Palm stress reduces DOC2B protein abundance without changing mRNA levels, we evaluated the DOC2B levels in cells when protein translation is inhibited with cycloheximide (CHX). The combination of CHX + Palm remarkably reduced DOC2B protein abundance, compared to that of Veh + CHX (Figure 3G). These results suggest that 24 h Palm exposure negatively affected DOC2B protein abundance, likely at a post-transcriptional/protein stability level.

### 2.4. DOC2B-Enrichment Promotes Insulin-Stimulated GLUT4 Translocation under Lipotoxic Conditions

**Figure 4:**
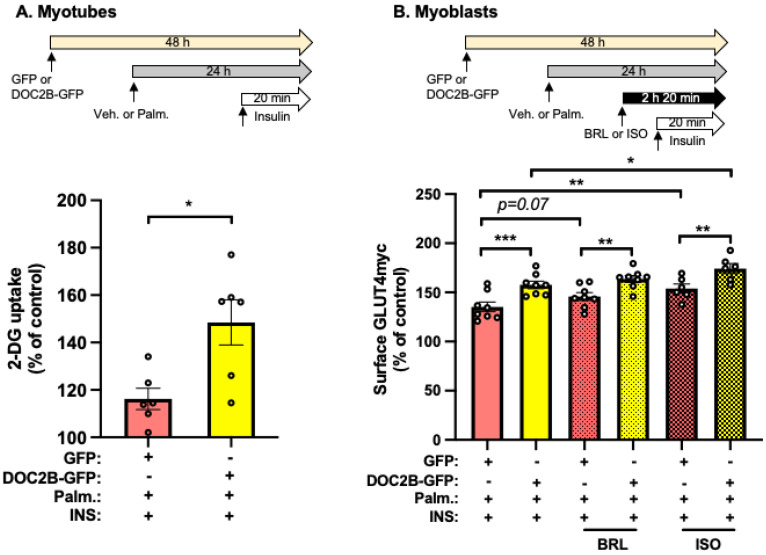
DOC2B enrichment in combination with β-AR agonism promotes insulin-stimulated glucose uptake and GLUT4 translocation under Palm-stress conditions. (**A**,**B**) Analysis of 2-DG uptake levels (**A**) and surface GLUT4myc levels (**B**) in control GFP or DOC2B-enriched L6-GLUT4myc muscle cells subjected to stated conditions (*n* = 6–8 independent experiments). Data are expressed as mean ± SEM. * *p* < 0.05, ** *p* < 0.01, *** *p* < 0.001.

Adenoviral-transduced myotubes expressing either rat DOC2B fused to GFP (DOC2B-GFP) to distinguish from endogenous DOC2B by immunoblot or GFP alone (control) [14] were used to investigate whether DOC2B-enrichment could protect against Palm-induced impairment of 2-DG uptake into myotubes. DOC2B enrichment protected against the negative effects of Palm on insulin-stimulated 2-DG uptake, as compared with the vector control (GFP) (Figure 4A) in L6 myotubes. Similarly, L6-GLUT4myc myoblasts transfected with DOC2B-GFP plasmid displayed ameliorated Palm stress impairment of GLUT4myc accumulation at the PM, compared with vector control (Figure 4B). DOC2B-GFP enrichment did not alter the endogenous DOC2B protein levels (Supplemental Figure S1). Moreover, ISO addition to DOC2B-GFP expressing cells further improved insulin-stimulated GLUT4myc accumulation at the PM under Palm-induced stress conditions (Figure 4B). These data suggest that the collaboration between DOC2B and the β-AR signaling pathway is beneficial in preventing Palm-induced dysfunction in insulin-stimulated GLUT4 vesicle exocytosis.

### 2.5. DOC2B Enrichment Protects against Palm-Induced Stress by Attenuating Phosphorylated Cofilin Levels

**Figure 5:**
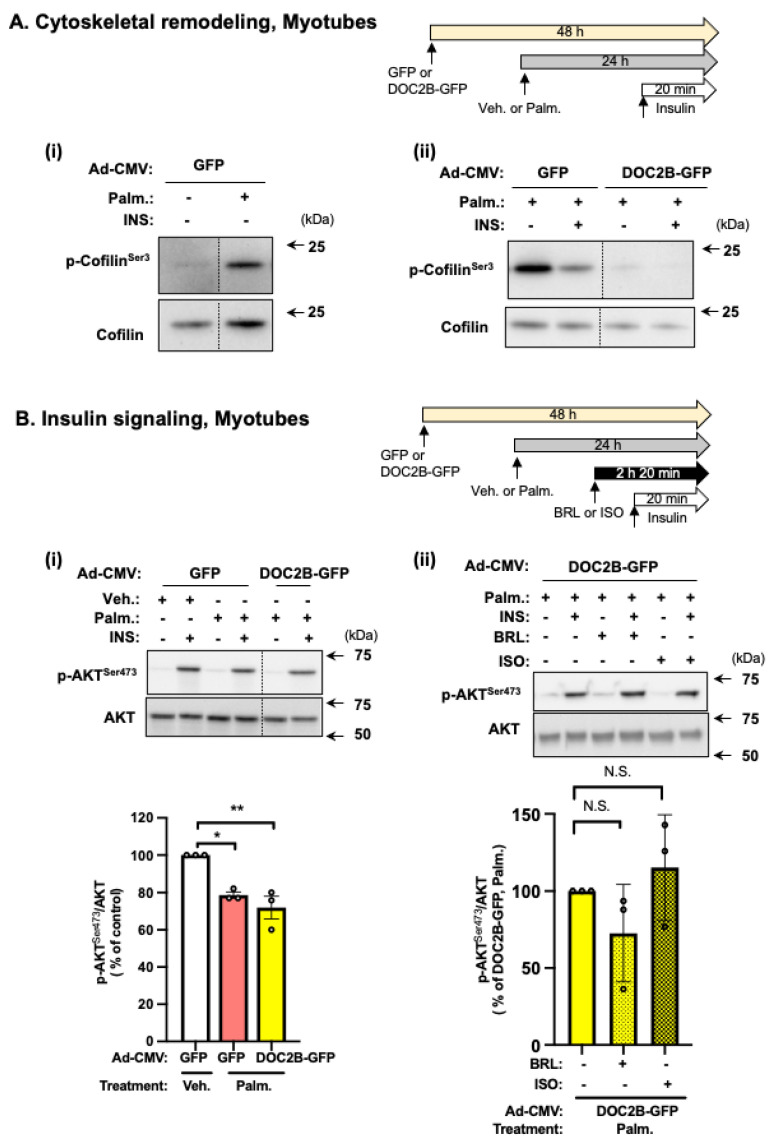
DOC2B protects against Palm-induced cofilin phosphorylation in L6-GLUT4myc myotubes. (**A**) Effect of adenoviral transduced DOC2B enrichment (or GFP alone) ± insulin (INS) stimulation on p-Cofilin^Ser3^ under Palm-stress (or Veh.) conditions. A representative immunoblot is shown (*n* = 4 independent experiments). (**B**) Effect of adenoviral transduced DOC2B enrichment (or GFP alone) and ± insulin (INS) stimulation on downstream insulin signaling effector p-AKT^Ser473^ under Palm-stress (or Veh.) conditions. Under some conditions, the added effect of β-AR agonism (BRL or ISO) was analyzed. A representative immunoblot is shown (*n* = 3 independent experiments). Bar graphs show densitometric analysis of phosphorylated AKT^Ser473^ normalized to total AKT. Black vertical dashed lines indicate the splicing of lanes from within the same gel exposure. (**A**,**B**) Ponceau S staining of the full-length PVDF membrane was used as a loading control. Data are expressed as mean ± SEM. N.S.; not significant, * *p* < 0.05, ** *p* < 0.01.

We next sought to determine how DOC2B overexpression prevented Palm-stress-induced dysfunction in GLUT4 vesicle exocytosis. DOC2B regulates cytoskeletal remodeling in skeletal muscle and other cells [14,23,24], although the molecular step overseen by DOC2B remains unresolved. Since insulin-stimulated cytoskeletal remodeling is required for GLUT4 vesicle translocation to the PM [31,32], we used our Palm-stress-induced paradigm in L6-GLUT4myc myotubes to discern whether Palm stress induces cofilin phosphorylation (p-Cofilin^Ser3^). Induction of p-Cofilin^Ser3^ was previously reported to block cytoskeletal remodeling-facilitated GLUT4 vesicle translocation to the PM in myoblasts [27]. Palm stress markedly increased p-Cofilin^Ser3^ levels (Figure 5(Ai)); insulin-induced dephosphorylation of Cofilin is impaired by Palm (Figure 5(Aii)). No significant differences in total cofilin protein were detected across 4 independent passages of cells (Figure 5(Ai,Aii); *p* > 0.05). Remarkably, myotubes transduced to express DOC2B-GFP showed significantly abolished p-Cofilin^Ser3^ under all treatment conditions (Figure 5(Aii): the ratio of phosphorylated cofilin/total cofilin, from cells expressing GFP and exposed to Palm (+), for conditions INS (−) vs. INS (+), is 100 vs. 74.7 ± 12.7, respectively; *p* < 0.05, *n* = 4. GFP+ Palm+ INS− ratio was set equal to 100%, and GFP+ Palm+ INS+ values within the same independent passage of cells were normalized thereto. Data represent the mean ± SE of 4 independent passages of cells).

In contrast, DOC2B enrichment did not preserve insulin signaling, which was determined via the levels of phosphorylated AKT to test canonical AKT-dependent pathway under any conditions tested (Figure 5B). While our paradigm demonstrated the Palm-induced reduction of insulin-induced phosphorylation of AKT^Ser473^ in Veh-treated GFP L6-GLUT4myc myotubes as reported previously [33], DOC2B-GFP expressing cells were not protected from the Palm-stress (Figure 5(Bi)); the addition of BRL or ISO also failed to protect from Palm-induced stress (Figure 5(Bii)). Thus, the beneficial action of DOC2B and BRL or ISO in preventing Palm-induced dysfunction in insulin-stimulated GLUT4 vesicle exocytosis and glucose uptake is not mediated by preserving proximal insulin signaling in myotubes. Taken together, these data suggest that the protective effect of DOC2B is via its regulation of Cofilin phosphorylation, a distal event that is essential for GLUT4 translocation and subsequent glucose uptake.

## 3. Discussion

**Figure 6:**
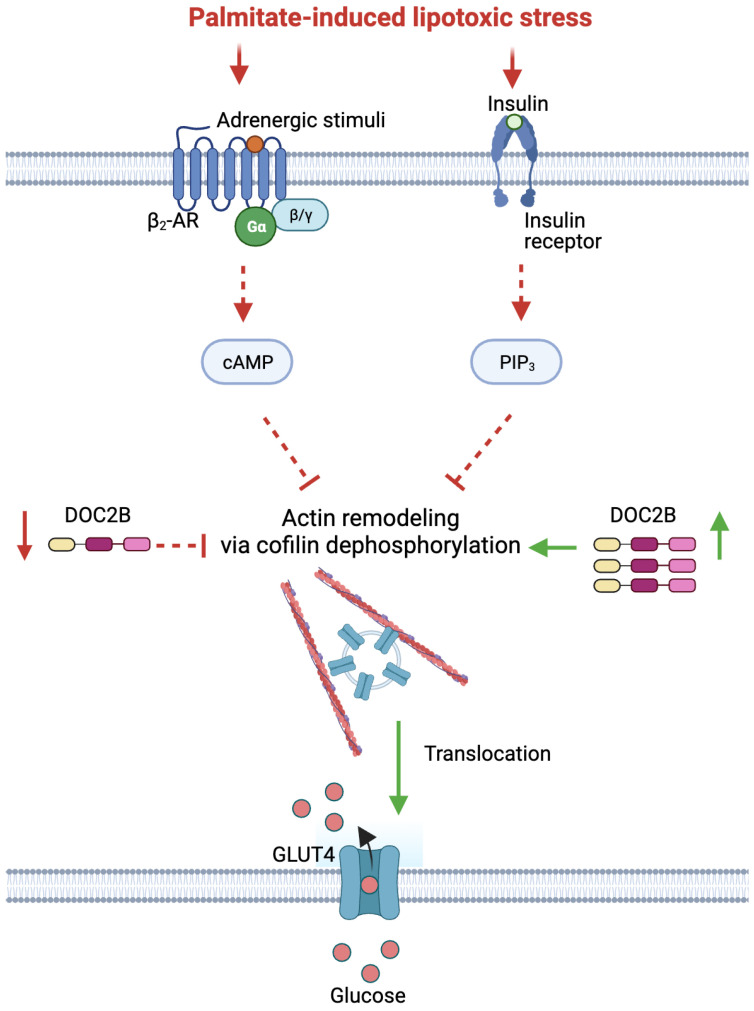
Schematic diagram of DOC2B-mediated GLUT4 translocation in response to β-AR agonists and insulin under palmitate-induced lipotoxic stress. Palmitate-induced lipotoxic stress results in a decrease in DOC2B expression, β-AR, and insulin signaling and an increase in cofilin phosphorylation, leading to reduced glucose uptake. Overexpressing DOC2B reverses this effect, triggering cofilin dephosphorylation. This process facilitates actin remodeling, ultimately restoring glucose uptake in myotubes. Red arrows indicate the negative effects of palmitate-induced lipotoxic stress. Green arrows indicate the positive changes associated with DOC2B overexpression.

In the current study, we generated a Palm-stress paradigm in L6-GLUT4myc myotubes and myoblasts that recapitulates the loss of DOC2B protein abundance observed in human T2D skeletal muscle [14], as well as the affiliated dysregulated GLUT4 vesicle exocytosis and glucose uptake. DOC2B enrichment protected against Palm-induced dysregulation of these processes and collaborated with β_2_-AR agonism to prevent dysregulation of insulin-stimulated GLUT4 accumulation at the PM and subsequent glucose uptake. Mechanistically, DOC2B enrichment was found to prevent Palm-induced cofilin phosphorylation, permitting cytoskeletal remodeling needed to build tracks upon which GLUT4 vesicles traffic to the PM [31,32], downstream and independent of insulin/β-AR signaling. We showed that by enhancing/stabilizing DOC2B levels in skeletal myotubes and myoblasts, the deleterious effects of Palm-exposure on cofilin, insulin, and β-AR-stimulated GLUT4 trafficking to the PM and glucose uptake were preventable (Figure 6).

While DOC2B upregulation in skeletal muscle has been previously shown to have a protective effect against high palmitate diet intake by mice (acute, 3 weeks feeding) [14], this benefit was attributed to the known role for DOC2B in SNARE-mediated exocytosis events at the distal steps of GLUT4 vesicle fusion. Intriguingly, proteomics analyses using healthy, non-Palm-stressed L6-GLUT4myc cells did reveal DOC2B to be a microtubule interacting protein via its interaction with the adaptor protein KLC1 [14]. DOC2B interaction with cofilin was not detected, perhaps because (1) DOC2B interacts with phosphorylated cofilin only, (2) additional proteins bridge DOC2B to phosphorylated cofilin, or (3) DOC2B-mediated dephosphorylation of cofilin is disease-context dependent in cases such as Palm-induced stress. Cofilin, a pivotal regulator of the actin cytoskeleton, is dephosphorylated in response to insulin stimulation to trigger GLUT4 translocation to the PM in healthy skeletal muscle [27]. The remodeling of actin involves continuous cycles of actin polymerization, with the involvement of actin-nucleating proteins such as the Arp2/3 complex, and actin-depolymerizing factors like cofilin and gelsolin [34,35]. Given that DOC2B both reduces cofilin phosphorylation and interacts with microtubule proteins, DOC2B may play a prominent role in coordinating actin and microtubule remodeling events that culminate in GLUT4 vesicle trafficking to the PM. Cofilin dephosphorylation is also a key outcome of metformin’s positive actions in lung endothelial cells, mediated via the protein phosphatase PP2AC [36]. The effects of metformin on DOC2B in the context of skeletal muscle remains to be elucidated. Future studies are needed to fully comprehend the cooperation between β-AR, DOC2B, and insulin and the downstream molecular mechanisms involved, including the signaling pathways associated with actin remodeling and microtubule activation mediated by DOC2B.

Based upon published work showing that β-AR activation in vivo could improve glucose tolerance in diabetic rodents [7,11], we anticipated that in our paradigm BRL/ISO would be able to mitigate Palm-induced dysfunction of GLUT4 accumulation at the PM of skeletal myotubes; however, no mitigation was detected. One interpretation for this difference is that the in vivo benefit of β-AR agonism arose from mechanisms beyond skeletal muscle GLUT4 accumulation and glucose uptake. Another distinction is that β-AR agonism in the in vivo study was chronic treatment, whereas our study used β-AR acutely for 2 h only. Regardless, our results do point to the relevance of β-AR agonism collaboration with DOC2B in preventing Palm-induced resistance to insulin in skeletal myotubes. This collaboration occurred distal to the phosphorylation of AKT since neither DOC2B nor β-AR prevented Palm-induced reduction of insulin signaling via phosphorylation of AKT. However, DOC2B enrichment with β-AR agonism did result in full prevention of Palm-induced loss of insulin-stimulated GLUT4 accumulation at the PM. DOC2B enrichment concurrent with β-AR agonism also enhanced insulin-stimulated GLUT4 accumulation at the PM under standard conditions (i.e., absence of Palm stress). This was a selective capability of DOC2B; upregulation of the sarcolemmal/t-tubule-localized GLUT4 vesicle docking and fusion protein Syntaxin 4 (STX4) failed to recapitulate the β-AR-enhancement effect seen with DOC2B upregulation (Supplementary Figure S2). Our data, which showed that Palm increased phosphorylated cofilin levels, support the concept proposed by Klip and colleagues [27] that increased cofilin levels and/or time cofilin are in a phosphorylated state keeps the F-actin in a state where it inhibits GLUT4 vesicle mobilization.

The distinction between DOC2B and STX4 may be twofold: (1) DOC2B was shown here to dephosphorylate cofilin, which is known to activate actin polymerization and formation of cytoskeletal tracks to the PM. By contrast, STX4 is not described as a cytoskeletal modifier in skeletal muscle cells. In islet beta cells, STX4 is implicated in depolymerizing cortical actin via interaction with the F-actin capping protein gelsolin; (2) unlike STX4, DOC2B is not anchored via a transmembrane domain allowing DOC2B to interact with non-PM proteins/networks away from the PM. STX4 carries a C-terminal transmembrane domain and in skeletal muscle cells this anchors STX4 at the PM to facilitate the tethering/docking and fusion of GLUT4 vesicles that have made their way to the PM [4], an event downstream of cytoskeletal remodeling and GLUT4 vesicle trafficking. STX4 is also present at the outer mitochondrial membrane in skeletal muscle cells [37], but this location is affiliated with a metabolic function, independent of its role in GLUT4 vesicle translocation. A third distinction between DOC2B and STX4 in this study was the significant vulnerability of DOC2B protein to Palm-induced stress, whereas STX4 was unimpacted (Supplemental Figure S3, [38]). Our observation was consistent across human and mouse primary muscle, and L6-GLUT4myc myotubes and myoblasts. Insulin action occurs in a fiber-type specific manner, and fiber-type specific differences in GLUT4 accumulation affect the development of insulin resistance [39,40,41]. Future studies will be needed to evaluate DOC2B protein abundance changes with obesity, pre-T2D, T2D, and Palm-based HFD and discern fiber-type- and muscle depot-related differences.

## 4. Materials and Methods

### 4.1. Animals, Diet, and Harvesting of Skeletal Muscle

The experimental protocols for this study were approved by the Institutional Animal Care and Use Committee at City of Hope (Duarte, CA, USA; approval #15023), according to the guidelines for the Care and Use of Laboratory Animals. For β-AR analysis, quadricep skeletal muscle was harvested from male C57BL/6J mice (20–22 weeks old) maintained on a standard chow diet (13% of kcal from fat; PicoLab #5053); the mice were fasted for 6 h prior to muscle harvest. For HFD intervention, male C57BL/6J mice (25 ± 0.6 g, 7–9 weeks old) were fed an HFD (45% calories from fat, palm oil-based; Research Diets, cat# D01030108i) ad libitum for 13 weeks to induce obesity-associated insulin resistance. Control male littermate mice were fed a standard chow diet ad libitum at the end of the feeding studies to provide a normal insulin sensitivity comparison group. All HFD-fed and control mice were housed individually on Sani-Chip bedding. Food was measured and changed twice a week, and body weight was measured once a week. After 13 weeks of HFD feeding, the quadriceps of skeletal muscle were harvested for protein analysis.

### 4.2. Cell Culture, Transient Transfection, and Adenoviral Transduction

Rat L6-GLUT4myc myoblasts stably expressing GLUT4 were purchased from Kerafast (Boston, MA, USA). Prior literature showed no GLUT4 expression in L6 myoblasts until the myotube differentiation [42]. To overcome this limitation, Klip and colleagues engineered L6 myoblasts expressing a myc epitope-tagged GLUT4 called L6-GLUT4myc myoblasts [43] in which the c-Myc epitope (14 amino acids) was strategically targeted to the first exofacial loop of GLUT4 without compromising its functionality [44]. Myoblasts (4 × 10^4^ cells/mL) were grown in Minimum Essential Medium-a (MEM-α) containing 5.5 mM glucose supplemented with 10% FBS (vol./vol.) and 1% (vol./vol.) antibiotic-antimycotic solution (Thermo Fisher, Waltham, MA, USA), as described previously [14]. The cells were maintained at less than 70% confluency to avoid spontaneous differentiation. For differentiation of myoblasts into myotubes, L6-GLUT4myc myoblasts (1 × 10^4^ cells/mL) were placed into MEM-α medium containing 5.5 mM glucose supplemented with 2% FBS (vol./vol) and 1% (vol./vol.) antibiotic-antimycotic solution for 7 days at 37 °C, 5% CO_2_, and the medium was refreshed every other day. The differentiation status of L6-GLUT4myc myoblasts was regularly monitored under a microscope. For expression of the target gene of interest (DOC2B-GFP or control plasmid DNA), myoblasts were transfected with 0.5 μg of plasmid DNA per well of a 24-well plate using L6 Cell Avalanche™ Transfection Reagent (EZ Biosystems, College Park, MD, USA, cat# EZT-L600-1) for 6 h according to the manufacturer’s protocol. Myotubes were transduced for 24 h with either AdCMV-rat (r) DOC2B-GFP or AdCMV-GFP (Control) virus (ViraQuest, North Liberty, IA, USA) at MOI = 100. Transductions were performed as previously described [14].

### 4.3. RNA Extraction and Quantitative Real-Time qPCR Analysis (RT-qPCR)

Total RNA was isolated using an RNeasy Plus Mini kit (Qiagen, Hilden, Germany, cat# 74136) for cells and a Tri Reagent^®^ for tissues according to the manufacturer’s instructions. For L6-GLUT4myc myotubes, complementary DNA was prepared using the iScript cDNA synthesis kit (Bio-Rad (Paramount, CA, USA), cat# 1708891), and the qPCR analysis was performed using the iQ™ SYBR^®^ Green Supermix (Bio-Rad, cat# 1708882). For myoblast and tissues, 50 ng of RNA/reaction was used for real time-qPCR using the QuantiTect SYBR Green RT-PCR Kit (Qiagen, cat# 204243) under the following conditions: 40 cycles, at 94 °C for 15 s, 58 °C for 30 s, and 72 °C for 30 s. Relative gene expression was normalized to HPRT using the ∆∆CT method. The primer sequences used are shown in Supplementary Table S1.

### 4.4. Pharmacologic Intervention

For pharmacologic intervention, 6 h-post-transfected L6-GLUT4myc myoblasts (rDOC2B-GFP or GFP; see above) or 24 h-post-transduced myotubes (AdCMV-rDOC2B-GFP or AdCMV-GFP; see above) were washed 3x with PBS, and then further cultured for 48 h. To test for synergistic effects of DOC2B with β-AR agonists (BRL-37344, R&D Systems (Minneapolis, MN, USA), cat# 0948; Isoproterenol, Sigma (St. Louis, MO, USA), 5984-95-2), cells were treated with BRL-37344 (10 µM) or isoproterenol (1 µM) for 2 h in serum-free medium and were then stimulated with/without insulin (100 nM) for 20 min. To validate the specificity of the additive effect of the agonists, cells were pretreated with the β-AR blocker, propranolol (10 μM), for 30 min before the addition of BRL or ISO, and then stimulated with insulin for 20 min. To investigate DOC2B stability, L6-GULT4myc myoblasts were treated with cycloheximide (CHX) (final 50 µg/mL) (Sigma-Aldrich (St. Louis, MO, USA), cat# C4859) in the presence or absence of Palm (200 µM) for 24 h (see below).

### 4.5. Cell-Surface GLUT4myc Detection

Cell-surface GLUT4myc detection was performed as described previously [14,28]. This assay was conducted with non-permeabilized myoblasts. Briefly, L6-GLUT4myc myoblasts were serum-starved for 2 h prior to treatment with/without insulin (100 nM) for 20 min at 37 ˚C. The cells were washed 3× with ice-cold PBS to stop the reaction, fixed with 4% (wt/vol) paraformaldehyde in PBS for 30 min at room temperature, and then blocked in Odyssey blocking buffer (LI-COR Biosciences, Lincoln, NE, USA) for 1 h at room temperature. The cells were incubated with mouse anti-Myc (1:400) (Abcam (Cambridge, UK), cat# ab32) overnight at 4 °C, washed 3× with PBS, and then incubated with IRDye^®^ 800CW goat anti-mouse secondary antibody (1:750) (LI-COR Biosciences, cat# 926-32210) for 1 h at room temperature. The immunofluorescence intensity of the exofacial myc epitope was quantified using the Odyssey CLx infrared imaging system (LI-COR Biosciences), and the data were normalized to SYTO 60 (Invitrogen, Carlsbad, CA, USA), a red fluorescent nucleic acid stain.

### 4.6. Palmitate Stress Induction

Palm solution was prepared by conjugating palmitate to bovine serum albumin (BSA), as previously described [45], but with slight modifications. In brief, sodium palmitate (Sigma-Aldrich, cat# P9767) was dissolved in 0.01 M NaOH by heating at 70 °C until the solution became clear. Then, palmitate was conjugated to a 10% fatty acid-free, low endotoxin BSA solution (Sigma-Aldrich, cat# A1595) at 37 ˚C overnight to generate a 5 mM palmitate stock solution. For the control, the same amount of 0.01 M NaOH was added to a 10% BSA solution and incubated overnight at 37 °C. To cause chronic hyperlipidemic stress, L6-GLUT4myc myoblasts or myotubes were incubated with a final palmitate concentration of 200 µM for 24 h in MEM-α supplemented with 10% or 2% FBS, respectively. To assess the effects of DOC2B enrichment, L6-GLUT4myc myoblasts (24 h post-transfection, see above) or myotubes (48 h post-transduction, see above) were used.

### 4.7. 2-Deoxyglucose Uptake

The 2-DG uptake assay was conducted as described previously [14,28]. Briefly, L6-GLUT4myc myotubes were incubated in oxygenated FCB buffer (125 mM NaCl, 5 mM KCl, 1.8 mM CaCl_2_, 2.6 mM MgSO_4_, 25 mM HEPES, 2 mM pyruvate, and 2% (wt/vol) BSA) for 30 min in advance of acute insulin stimulation (100 nM) for 20 min. Glucose uptake was initiated by adding 2-Deoxy-D-glucose, 2-[1,2-^3^H (N)] (0.055 µCi/µL) (PerkinElmer (Waltham, MA, USA) Cat# NET328A250UC). After the addition of Deoxy-D-glucose, 2-[1,2-^3^H (N)] for 5 min, we immediately removed the media and performed 3× washes with cold PBS. This step was undertaken to halt the reaction and eliminate any residual Deoxy-D-glucose, 2-[1,2-^3^H (N)]. Subsequently, 1 N NaOH was added to the cells, followed by resuspension in 5 mL of scintillation cocktail (Bio-safe2, Research Product International Corp., Mount Prospect, IL, USA). The radioactivity of glucose incorporation was determined using a liquid scintillation analyzer (PerkinElmer, Waltham, MA, USA). Data were normalized to protein concentration, as determined using the Bicinchoninic acid assay (BCA) assay (Thermo Fisher Scientific, cat# 23225).

### 4.8. Cell Viability Assay

L6-GLUT4myc myoblasts were seeded at a density of 2 × 10^4^ cells/well in 96-well plates one day prior to administration of Palm (see above). Eight wells were used for each group. Following treatment with Palm for 24 h, cells were washed 3× with PBS, CCK-8 (10 µL, Abcam, Cat# ab228554) was added to each well, and cells were incubated for 2 h at 37 °C. The absorbance of the formazan product was measured at 450 nm.

### 4.9. Immunoblotting

After interventions, harvested cells were subsequently lysed with 1% Nonidet P-40 lysis buffer (25 mM HEPES pH 7.4, 1% Nonidet P-40, 10% glycerol, 137 mM NaCl, 1 mM sodium vanadate, 50 mM NaF, 10 mM NaPP, 10 µg/mL aprotinin, 5 µg/mL leupeptin, 1 µg/mL pepstatin, and 1 mM phenylmethylsufonyl fluoride). Protein concentrations were determined using the BCA assay. Samples were boiled for 5 min with 6× Laemmli SDS sample buffer (Bio-Rad Scientific LLC, Paramount, CA, USA) at 100 ˚C. The proteins were resolved using 10% SDS-PAGE gels and transferred onto PVDF membranes. Following the transfer, the membranes were blocked in 5% skim milk in TBST (10 mM Tris-HCL, pH 7.4, 150 mM NaCl, and 0.1% Tween 20) for 1 h at room temperature. The membranes were incubated with the indicated primary antibodies for 2 h at room temperature or 4 °C overnight: DOC2B (Proteintech Group (Rosemont, IL, USA), cat# 20574-1-AP), DOC2B against the human DOC2b 96-119 aa peptide sequence (PSPGPSPARPPAKPPEDEPDA) (custom synthesized antibody), GLUT4 (Abcam, cat# ab654), p-Cofilin (Santa Cruz (Dallas, TX, USA), cat# sc-271923), cofilin (Cell Signaling (Danvers, MA, USA), cat# 3312), p-AKT (Cell Signaling, cat# 4060), AKT (Cell Signaling, cat# 2920), and HPRT (Abcam, cat# ab10479). Then, membranes were incubated for 1 h at room temperature with a horseradish peroxidase-conjugated goat anti-rabbit IgG (HL) (Bio-Rad, cat# 172-1019) or goat anti-mouse (HL) (Bio-Rad, cat# 172-1011). The specificity of the DOC2B antibody (custom) was confirmed using a DOC2B-specific blocking peptide. Chemiluminescence was documented using a Bio-Rad ChemiDoc Touch and ECL (Amersham ECL Western Blotting Detection Reagent, GE Healthcare (Chicago, IL, USA), cat# RPN2106). Densitometric analysis of bands of interest was performed using Image Lab software version 6.1(Bio-Rad). All experimental samples and controls used for one comparative analysis were run on the same blot.

### 4.10. Statistical Analysis

Data are expressed as the means ± standard error of the mean (SEM). Data were evaluated for statistical significance using a two-tailed, unpaired Student’s *t*-test for comparison of two groups. One-or two-way analysis of variance (ANOVA) followed by post poc Dunnett or Tukey post hoc test was used for more than two groups using GraphPad Prism Version 9.0 (GraphPad Software, La Jolla, CA, USA). Significance was determined when the *p*-value was ≤0.05.

## 5. Conclusions

In conclusion, we revealed here a useful paradigm of Palm-induced stress to test for factors that can prevent/reverse skeletal muscle dysfunctions related to obesity/pre-T2D. This paradigm induced insulin- and β-AR resistance and reduced DOC2B protein levels, which in turn led to deregulated cofilin phosphorylation and reduced GLUT4 levels at the PM. By enhancing DOC2B levels in skeletal myotubes and myoblasts, we showed that the deleterious effects of palmitate exposure upon cofilin, insulin, and β-AR-stimulated GLUT4 trafficking to the PM and glucose uptake were preventable. Discerning strategies to upregulate or stabilize DOC2B protein levels in skeletal muscle could provide avenues to prevent and/or reverse skeletal muscle insulin resistance and halt progression to pre-T2D and T2D.

## Data Availability

The data presented in this study are available on request from the corresponding author.

## Supplementary Materials

The following supporting information can be downloaded at: https://www.mdpi.com/article/10.3390/ijms25010137/s1.
