# Supplementary material for: A Novel Role for DOC2B in Ameliorating Palmitate-Induced Glucose Uptake Dysfunction in Skeletal Muscle Cells via a Mechanism Involving β-AR Agonism and Cofilin
Source: Int J Mol Sci. 2023 Dec 21;25(1):137. doi: 10.3390/ijms25010137 (PMC10779393; doi:10.3390/ijms25010137)
Supplement: Supplementary file 1 [file ijms-25-00137-s001.zip › ijms-2740502-supplementary.pdf]

SUPPLEMENTAL MATERIALS

A novel role for DOC2B in ameliorating palmitate-induced glucose uptake dysfunction in skeletal muscle cells, via a mechanism involving β-AR agonism and cofilin

Jinhee Hwang[1,2], Rekha Balakrishnan[1], Eunjin Oh[1], Rajakrishnan Veluthakal[1], and Debbie C. Thurmond[1]*

1 Department of Molecular and Cellular Endocrinology, Arthur Riggs Diabetes and Metabolism Research Institute, Beckman Research Institute at City of Hope, Duarte, CA 91010, USA; jihwnag@korea.ac.kr (J.H.); rbalakrishnan@coh.org (R.B.); euoh@coh.org (E.O.); rveluthakal@coh.org (R.V.)

2 Department of Food and Biotechnology, College of Science and Technology, Korea University, Sejong 30019, Republic of Korea

*** Correspondence: dthurmond@coh.org; Tel.: +1-626-218-0190; Fax: +1-626-256-8704**

TABLE OF CONTENTS

Supplementary Table S1 2
Supplemental Figure S1 3
Supplemental Figure S2 4
Supplemental Figure S3 5
Supplementary Methods 6
Supplemental reference: 8

Supplementary Table S1.
Primer sequences used for quantitative real-time PCR.

Species	Gene	Forward primer (5’ to 3’)	Reverse primer (5’ to 3’)
Mouse	DOC2B	CCAGCAAGGCAAATAAGCTC	ATTGGGCTTCAGCTTCTTCA
	ADRB1	AGACCGAAAGCAGGTGAATG	ACTCTCCCAACTCCTCCTAAA
	ADRB2	ACTCTGCCTTCAATCCTCTTATC	GTAGCCGTTCCCATAGGTTT
	ADRB3	TCCCTTTCTTCCTACTGCTTTC	CTGGATTCCTGCTCTCAAACT
	HPRT	AAGCCTAAGATGAGCGCAAG	TTACTAGGCAGATGGCCACA
Rat	DOC2B	CCAGCAAGGCAAATAAGCTC	GTTGGGTTTCAGCTTCTTCA
	ADRB1	CGTCCGTCGTCTCCTTCTA	GCAGCTGTCGATCTTCTTCA
	ADRB2	GCTATCGCTTCCTCTATCGTATC	GCCCTCGGATTTGTCTATCTT
	ADRB3	GCTGTTCCTTTGCCTCCAATA	GAGCATAGACGAAGAGCATCAC
	HPRT	GGTCCATTCCTATGACTGTAGATTTT	CAATCAAGACGTTCTTTCCAGTT

Supplemental Figure S1

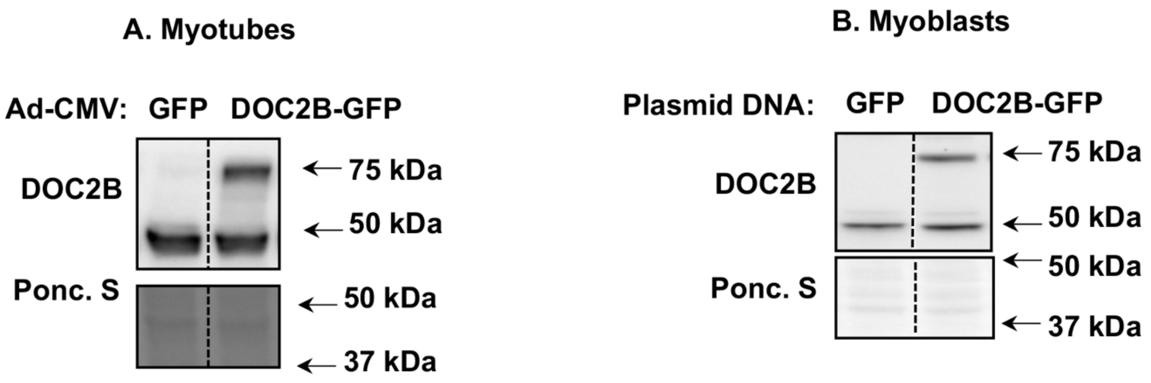

Figure S1: Validation of DOC2B overexpression in L6-GLUT4myc cells. A-B) Immunoblot analysis of DOC-GFP expression in control GFP or DOC2B-GFP adenovirus-transduced L6-GLUT4myc myotubes (A) and in control GFP or DOC2B-GFP plasmid DNA-transfected L6-GLUT4myc myotubes (B). A representative immunoblot is shown. Black vertical dashed lines indicate splicing of lanes from within the same gel exposure. Ponceau S staining of the full length PVDF membrane was used as a loading control.

Supplemental Figure S2

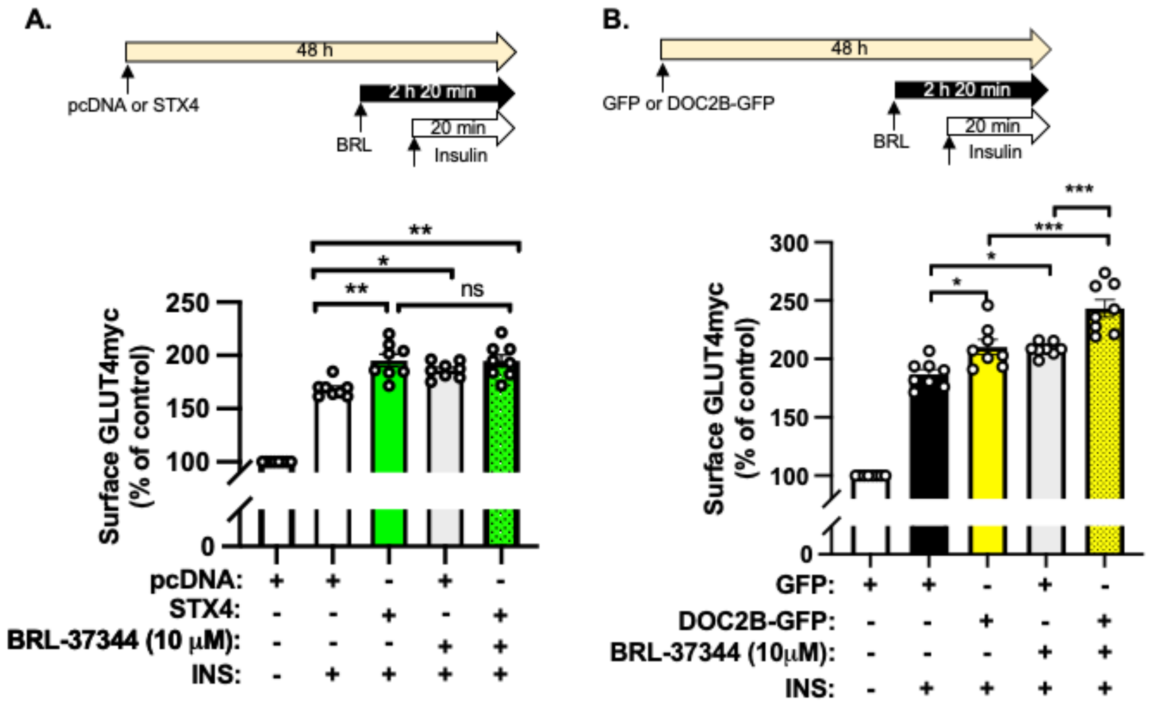

Figure S2: **Effect of Syntaxin 4 (STX4) enrichment with β-AR agonism on insulin-stimulated GLUT4 translocation in L6 myoblasts. (A-B)** L6-GLUT4myc myoblasts were transfected with indicated vector (STX4 vs. pcDNA [control] or DOC2B-GFP vs. GFP [control]) were treated with ± β-AR agonist (BRL-37344) and then stimulated with ± insulin (INS). **(A)** Quantification of GLUT4myc levels at the plasma membrane (PM) with ± STX4 enrichment under stated conditions (n=8 independent experiments). **(B)** Quantification of GLUT4myc levels at the PM with ± DOC2B enrichment under stated conditions (n=8 independent experiments). N.S., Not Significant. *p<0.05, **p<0.01, ***p<0.001.

Supplemental Figure S3

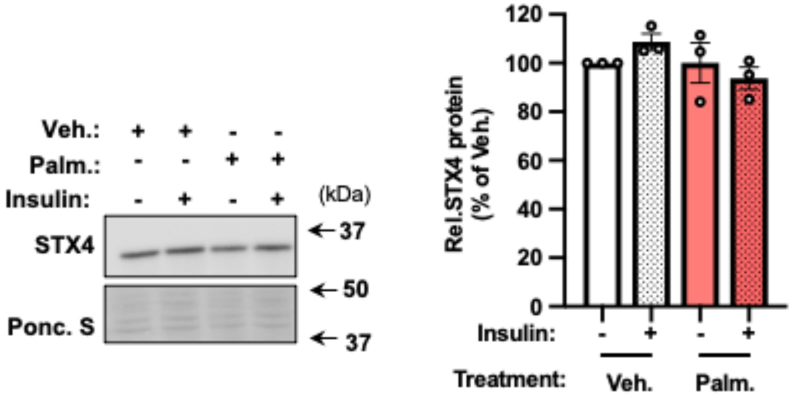

Figure S3: **Effect of Palm-stress on STX4 protein abundance.** Immunoblot analysis and quantification of STX4 protein abundance in L6-GLUT4myc myotubes ± palmitate (Palm)-stress (n=3 independent experiments). A representative immunoblot is shown. Ponceau S (Ponc. S) staining of the full length PVDF membrane was used as loading control.

Supplementary Methods

Overexpression DOC2B or STX4 and pharmacologic intervention - The L6-GLUT4myc myoblasts at 70% confluence were transfected with 0.5 μg of rDOC2B-GFP or pcDNA-rSTX4, or the control plasmid DNA (GFP or pcDNA) in 24-well plates using L6 Cell Avalanche™ Transfection Reagent (EZ biosystems, MD, USA, cat# EZT-L600-1) for 6 h according to the manufacturer's protocol, washed with PBS to remove any free complex, and then further cultured for 48 h. To test for synergistic effects of DOC2B or STX4 with β_2/β_3-adrenergic receptor agonist (BRL-37344, R&D Systems, cat# 0948), cells were treated with BRL-37344 (10 μM) for 2 h in serum-free medium and were then stimulated with or without insulin (100 nM) for 20 min. Palm-induced stress was carried out by treating L6-GLUT4myc myoblasts with 200 μM of Palmitate for 24 h after the initial 24 h of transfection.

Immunoblotting - After Palmitate or BSA treatment for 24 h, L6-GLUT4myc myotubes were subsequently lysed with 1% Nonidet P-40 lysis buffer (25 mM HEPES pH 7.4, 1% Nonidet P-40, 10% glycerol, 137 mM NaCl, 1 mM sodium vanadate, 50 mM NaF, 10 mM NaPP, 10 μg/ml aprotinin, 5 μg/ml leupeptin, 1μg/ml pepstatin, and 1 mM phenylmethylsufonyl fluoride). Protein concentrations were determined by the BCA assay. Samples were boiled for 5 min with 6x Laemmli SDS sample buffer (Bioland Scientific LLC, Paramount, CA, USA) at 100˚C. The proteins were resolved by 10% SDS-PAGE gels and transferred onto PVDF membranes. Following the transfer, the membranes were blocked in 5% skim milk in TBST (10 mM Tris-HCL, pH 7.4, 150 mM NaCl, and 0.1% Tween 20) for 1 h at room temperature. The membranes were incubated with the indicated primary antibodies for 2 h at room temperature or at 4˚C overnight: STX4 (custom synthesized as described [1], 1:5000). Then, membranes were incubated for 1 h at

room temperature with a horseradish peroxidase-conjugated goat anti-rabbit IgG (HL) (Bio-Rad, cat#172-1019) or goat anti-mouse (HL) (Bio-Rad, cat # 172-1011). Chemiluminescence was documented using a Bio-Rad ChemiDoc Touch and ECL (Amersham ECL Western Blotting detection reagent, GE Healthcare, cat# RPN2106). Densitometric analysis of bands of interest was performed using Image Lab software (Bio-Rad).

Supplemental reference:

1. Wiseman, D.A.; Kalwat, M.A.; Thurmond, D.C. Stimulus-induced S-nitrosylation of syntaxin 4 impacts insulin granule exocytosis. *J Biol Chem* **2011**, *286*, 16344-16354, doi: 10.1074/jbc.M110.214031.
